# A new acidophilic thermostable endo-1,4-β-mannanase from *Penicillium oxalicum* GZ-2: cloning, characterization and functional expression in *Pichia pastoris*

**DOI:** 10.1186/s12896-014-0090-z

**Published:** 2014-10-28

**Authors:** Hanpeng Liao, Shuixian Li, Haiping Zheng, Zhong Wei, Dongyang Liu, Waseem Raza, Qirong Shen, Yangchun Xu

**Affiliations:** Jiangsu Collaborative Innovation Center for Solid Organic Waste Utilization, College of Resources and Environmental Science, Nanjing Agricultural University, Nanjing, 210095 China

**Keywords:** Endo-1,4-β-mannanase, Gene cloning, Expression system, *Pichia pastoris*, *Penicillium oxalicum*

## Abstract

**Background:**

Endo-1,4-β-mannanase is an enzyme that can catalyze the random hydrolysis of β-1, 4-mannosidic linkages in the main chain of mannans, glucomannans and galactomannans and has a number of applications in different biotechnology industries. *Penicillium oxalicum* is a powerful hemicellulase-producing fungus (Bioresour Technol 123:117-124, 2012); however, few previous studies have focused on the cloning and expression of the endo-1,4-β-mannanase gene from *Penicillium oxalicum*.

**Results:**

A gene encoding an acidophilic thermostable endo-1,4-β-mannanase (E.C. 3.2.1.78) from *Penicillium oxalicum* GZ-2, which belongs to glycoside hydrolase family 5, was cloned and successfully expressed in *Pichia pastoris* GS115. A high enzyme activity (84.4 U mL^−1^) was detected in the culture supernatant. The recombinant endo-1,4-β-mannanase (rPoMan5A) was tagged with 6 × His at its C-terminus and purified using a Ni-NTA Sepharose column to apparent homogeneity. The purified rPoMan5A showed a single band on SDS-PAGE with a molecular mass of approximately 61.6 kDa. The specific activity of the purified rPoMan5A was 420.9 U mg^−1^ using locust bean gum as substrate. The optimal catalytic temperature (10 min assay) and pH value for rPoMan5A are 80°C and pH 4.0, respectively. The rPoMan5A is highly thermostable with a half-life of approximately 58 h at 60°C at pH 4.0. The *K*_*m*_ and *V*_*max*_ values for locust bean gum, konjac mannan, and guar gum are 7.6 mg mL^−1^ and 1425.5 μmol min^−1^ mg^−1^, 2.1 mg mL^−1^ and 154.8 μmol min^−1^ mg^−1^, and 2.3 mg mL^−1^ and 18.9 μmol min^−1^ mg^−1^, respectively. The enzymatic activity of rPoMan5A was not significantly affected by an array of metal ions, but was inhibited by Fe^3+^ and Hg^2+^. Analytical results of hydrolytic products showed that rPoMan5A could hydrolyze various types of mannan polymers and released various mannose and manno-oligosaccharides, with the main products being mannobiose, mannotriose, and mannopentaose.

**Conclusion:**

Our study demonstrated that the high-efficient expression and secretion of acid stable and thermostable recombinant endo-1, 4-β-mannanase in *Pichia pastoris* is suitable for various biotechnology applications.

## Background

Mannan polysaccharides are the major components of softwood hemicellulose and can be classified into linear mannan, glucomannan, galactomannan, and galactoglucomannan [1]. They consist of a backbone of β-1,4-linked mannose, which may be interrupted by D-glucose in glucomannans. Due to the complex nature of these polysaccharides, a combination of different enzymes such as endo-1,4-β-mannanase (EC 3.2.1.78), β-mannosidase (EC 3.2.1.25), β-glucosidase (EC 3.2.1.21), α-galactosidase (EC 3.2.1.22) and acetyl mannan esterase (EC 3.1.1.6) are required to complete the hydrolysis of heteromannans. Among them, the most important is endo-1,4-β-mannanase (EC 3.2.1.78), which can catalyze random internal hydrolysis of β-1,4-mannosidic linkages in the main structure of β-1,4-mannans, glucomannans and galactomannans by releasing small manno-oligosaccharides [2]. This type of enzyme has shown a wide range of potential uses in industrial applications, including food/feed, pharmaceutical, pulp/paper, gas well stimulation [3], as well as for second generation biofuels [4,5]. Mannans, the major components of hemicellulose, associate with lignin and cellulose in lignocellulosic biomass, physically hindering cellulase access to the fibers and reducing the efficiency of enzymatic hydrolysis [6]. In the bioenergy process, the breakdown of lignocellulose to fermentable sugars by enzymatic hydrolysis is one of the decisive bottlenecks, due to the recalcitrance of the plant cell wall and the high cost of enzymes. Based on this application, these enzymes have recently received much attention for industrial use, with an ever-increasing demand for renewable bioenergy utilization.

During the past few decades, many endo-1,4-β-mannanases have been purified and characterized from plants, bacteria, fungi and metazoans, among which the filamentous fungi are considered to be good candidates for the industrial production of endo-1,4-β-mannanases [7]. Many studies have been performed focusing on discovering new endo-1,4-β-mannanases with robust enzymatic activity by various methods, such as mutating enzyme-producing strains and optimizing fermentation conditions, and on producing endo-1,4-β-mannanases by fermentation on an industrial scale [8,9]. However, a suitable endo-1,4-β-mannanase for industrial use involves not only a high enzymatic hydrolysis activity; but thermo- and acidic-stability are also needed as many industrial bioprocesses require the use of enzymes at low pH and high temperature conditions to prevent contamination by other microbes [10–12].

*P. oxalicum* GZ-2, an agricultural biomass-degrading filamentous fungus, was isolated in our lab from decaying-wood acidic soil and showed favorable acidophilic hemicellulase-producing ability [13]. When *P. oxalicum* GZ-2 was incubated with agricultural waste (corn cob) as carbon source, an obvious mannanase activity could be determined in the culture supernatant (data not shown). In this study, a new acidic thermostable GH5 endo-1,4-β-mannanase gene (*poman5A*) from *P. oxalicum* GZ-2 was cloned and heterologously expressed in *Pichia pastoris* GS115. To our knowledge, this is the first example of cloning and expression of an acidic thermostable endo-1,4-β-mannanase gene from *P. oxalicum*. The biochemical characteristics of the recombinant enzyme were also evaluated. The superior biochemical and hydrolytic properties of rPoMan5A make it a highly useful candidate for various industrial applications.

## Results

### Cloning and sequence analysis of the endo-1,4-β-mannanase gene from *P. oxalicum* GZ-2

**Figure 1:**
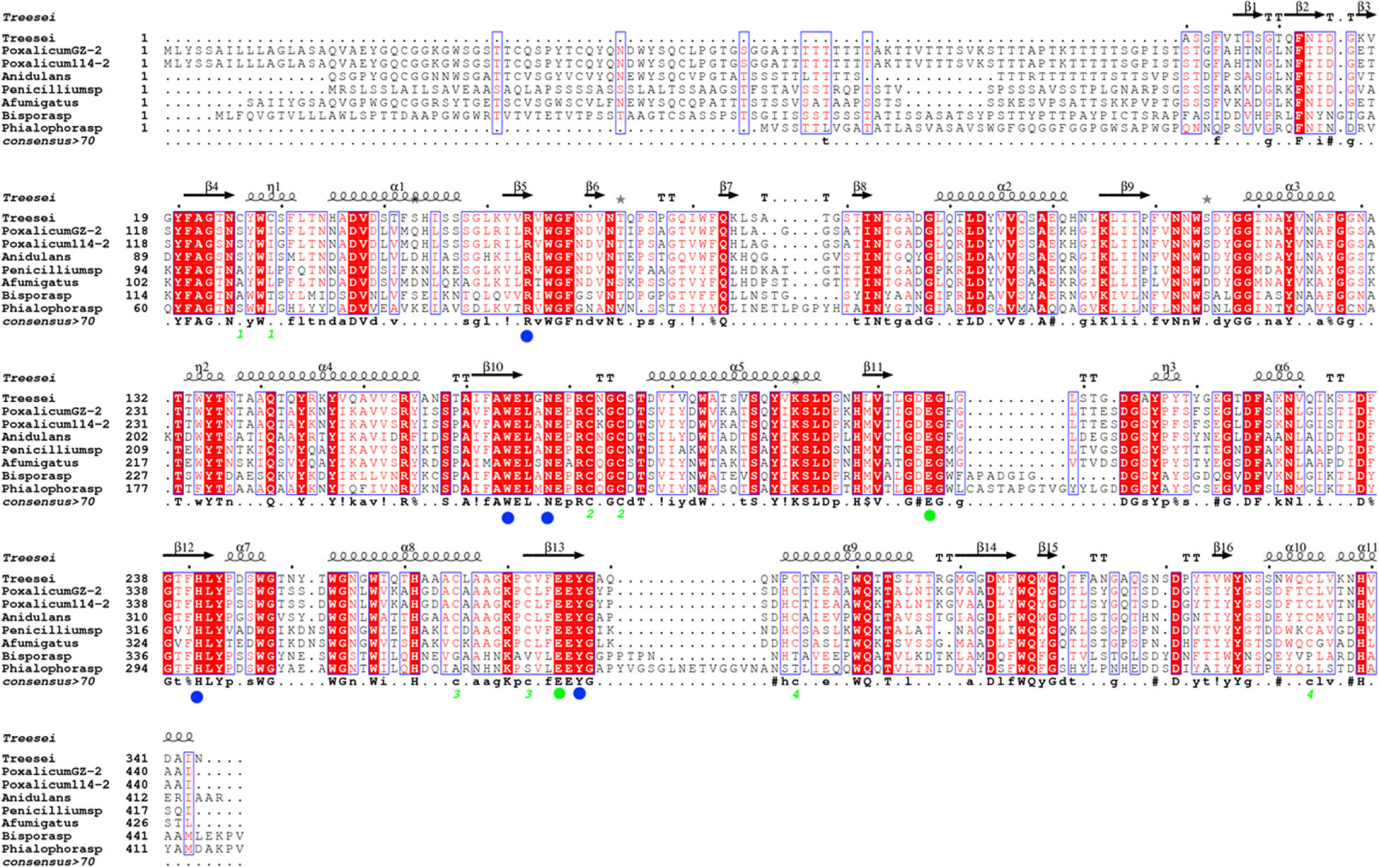
**Amino acid sequence alignment of endo-β-1, 4-mannosidase from** ***P. oxalicum*** **GZ-2 with related fungi.** The secondary structural elements (α-helices displayed as squiggles, β-strands rendered as arrows, and strict β-turns as TT letters) of rPoMan5A are using *Trichoderma reesei* β-mannanase as a template (pdb no. 1QNO_A). The alignment includes endo-β-1, 4-mannanases from *P. oxalicum* GZ-2 (GenBank: AGW24296.1), *Penicillium oxalicum* 114-2 (GenBank: EPS31069.1), *Penicillium* sp. (GenBank: AFC38441.1), *Bispora* sp. MEY-1 (GenBank: ACH56965.1), *Phialophora* sp. (GenBank: ADF28533.1), *Aspergillus fumigatus* (GenBank: ACH58410.1), *Aspergillus nidulans* (GenBank: AGG69666.1), and *Trichoderma reesei* QM6a (GenBank: XP_006962944). Strictly conserved residues are highlighted with a red background and conservatively substituted residues are boxed. Pairs of Cys residues that formed disulfide bonds are indicated by green-colored numbers. The conserved catalytic residues Glu320 and Glu391 are indicated by a green dot and other conserved amino acid residues are marked with a blue dot. The figure was made using ESPript 3.0.

A 772-bp gene fragment was amplified from the gDNA of strain GZ-2 using the degenerate primers manA-df and manA-dr. Sequence analysis indicated that the fragment showed 76% identity with an endo-1,4-β-mannanase from *Aspergillus aculeatus* (GenBank: L35487.1)*.* To amplify the full-length endo-1,4-β-mannanase gene the 5’ and 3’ flanking regions were obtained using self-formed adaptor PCR (SEFA-PCR). All fragments were assembled with core regions to create a 1671 bp sequence containing a complete chromosomal gene of 1380 bp predicted by FGENESH (http://linux1.softberry.com/berry.phtml). The full-length cDNA sequence of endo-1,4-β-mannanase (PoMan5A) was contained in an open reading frame of 1380 bp, which was cloned from the synthesized cDNA using the manA-f and manA-r primers. Three introns interrupted the PoMan5A coding sequence according to the alignment of the DNA and cDNA sequences. A putative 33-residue signal peptide at the N-terminus was predicted by the SignalP server system (http://www.cbs.dtu.dk/services/SignalP/). The deduced mature protein of PoMan5A contained of 426 residues with a calculated molecular mass of 45.8 kDa and a theoretical pI of 5.09 (http://web.expasy.org/compute_pi/). The deduced amino acid sequence of PoMan5A showed the highest identity of 100% with the putative endo-1,4-β-mannanase from *Penicillium oxalicum* 114-2 (EPS31069). PoMan5A contained an N-terminal fungal cellulose binding domain and a glycosyl hydrolase family 5 domain. After multiple sequence alignments with other GH5 endo-1,4-β-mannanase amino acid sequences, the two catalytic glutamates (Glu 320 and Glu 391) are located in highly conserved regions of the active site in PoMan5A. Several amino acid residues, Arg169, Trp279, Asn283, His357, and Tyr393 are conserved in PoMan5A and other fungal GH5 endo-β-1, 4-mannanases (Figure 1). The nucleotide sequence of *poMan5A* was deposited into GeneBank under accession number KF233753.

### Heterologous expression and purification of the *P. oxalicum* endo-1,4-β-mannanase

**Figure 2:**
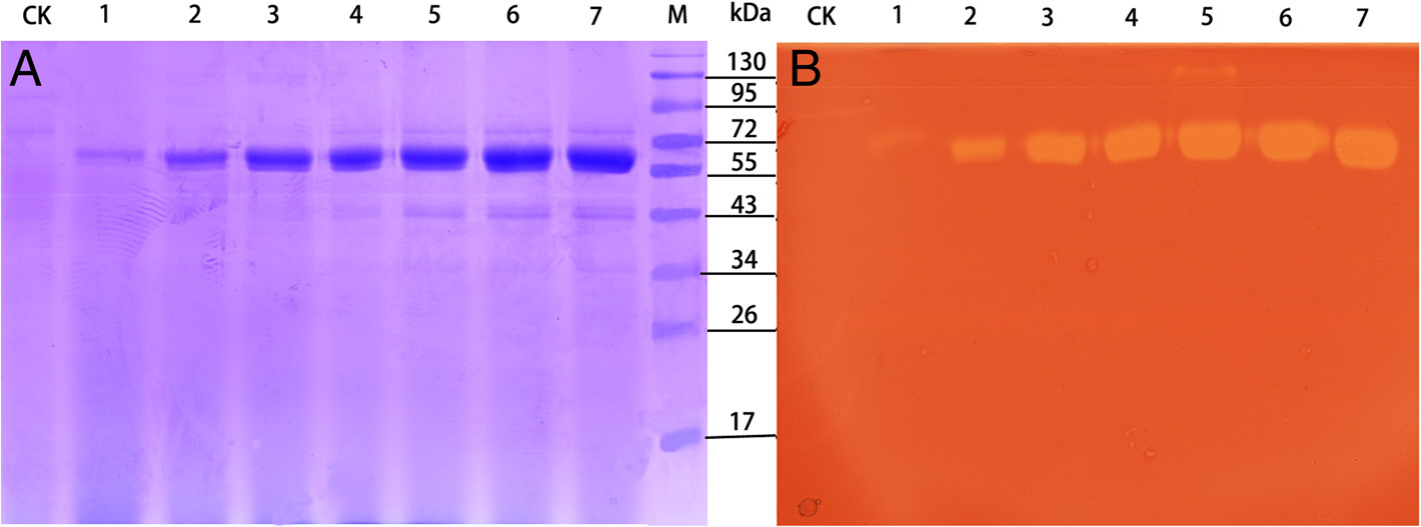
**SDS-PAGE and zymography analysis of culture supernatants from recombinant** ***Pichia pastoris*** **during 7-day fermentation.** SDS-PAGE **(A)** and zymography **(B)** of secreted proteins produced by recombinant *Pichia pastoris* fusing the *P. oxalicum* GZ-2 endo-β-1, 4-mannosidase gene. Lane CK: *Pichia pastoris* GS115 with the empty pPICZαA after induction for 4 days; Lane 1-7: culture supernatant after induction for 1, 2, 3, 4, 5, 6, and 7 days; Lane M: protein molecular mass makers. Twenty microliters of culture supernatants was added into each gel lane.

**Figure 3:**
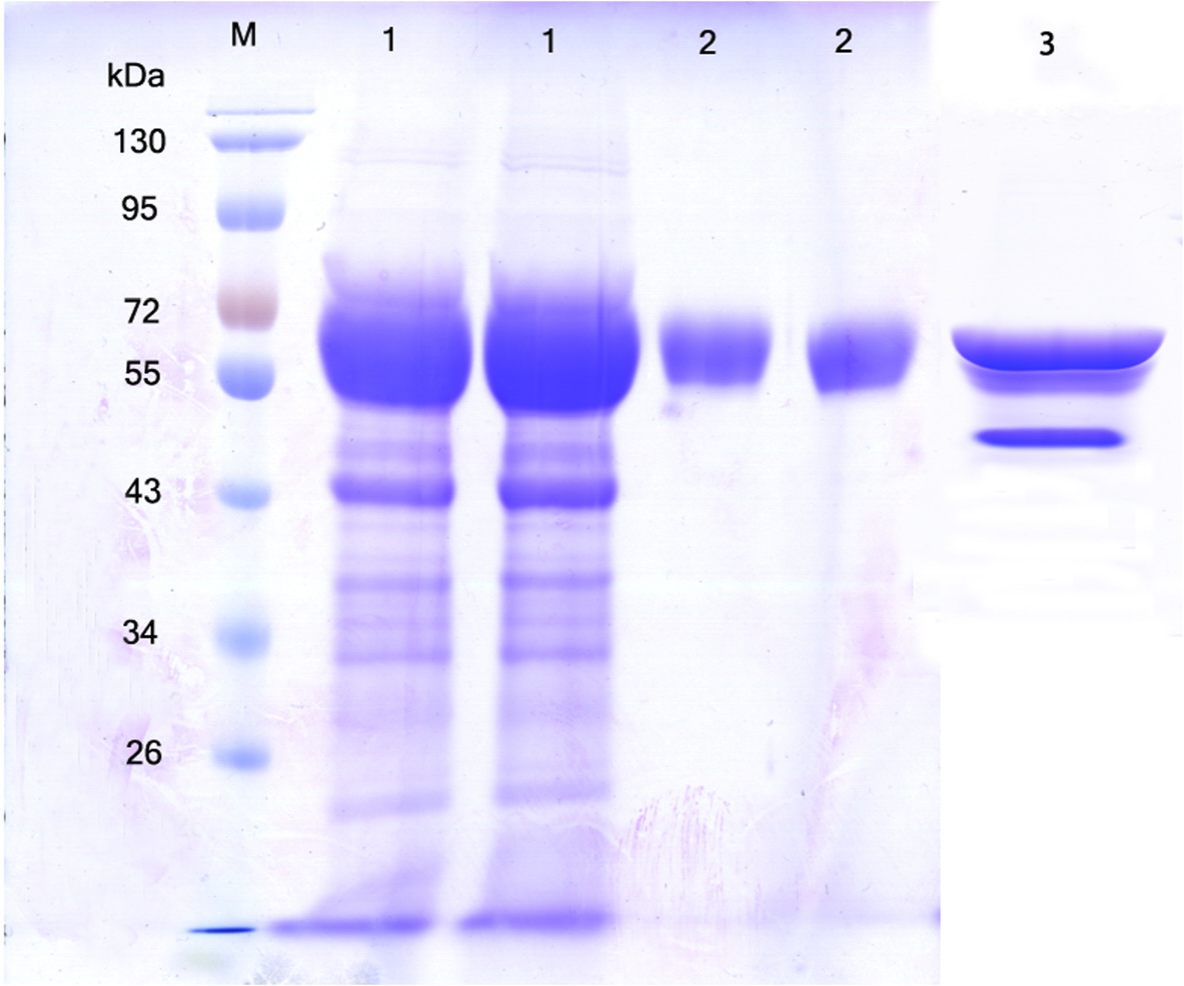
**SDS-PAGE analysis of the deglycosylation of rPoMan5A with Endo H**_**f**_ **and purified rPoMan5A fractions.** Lane 1, concentrated proteins from an ammonium sulfate precipitation; Lane 2, purified rPoMan5A; Lane 3, deglycosylated rPoMan5A after endo H_f_ treatment; Lane M, protein molecular mass makers.

The gene expression vector *pPIC-man5A* was successfully constructed for use in this study. In this recombinant plasmid, cDNA fragments were fused with the α-factor signal sequence at its N-terminus allowing secretion of enzyme into the liquid culture that was expressed under the control of the AOX1 promoter. The resulting plasmid was then transformed into *Pichia pastoris* by electroporation. After transformation and screening on plates containing Zeocin in increasing concentrations, 20 transformants showing robust antibiotic-resistance were further evaluated for their ability to secrete endo-1,4-β-mannanase in a small-scale liquid fermentation. One transformant with the highest enzyme activity was used to produce endo-1,4-β-mannanase in a 1 L Erlenmeyer flask induced by 1% methanol at 28°C for 7 days. The highest extracellular endo-1,4-β-mannanase activity (84.4 U mL^−1^) was obtained after a 6 day incubation at 28°C. SDS-PAGE analysis of the culture supernatants of the induction period from 1 to 7 day showed an apparent protein band (Figure 2A) corresponding to endo-1,4-β-mannanase activity in the zymogram analysis (Figure 2B). No endo-1,4-β-mannanase activity was detected in the culture medium of the control strain (transformed with pPICZαA) under the same culture conditions. The recombinant endo-1,4-β-mannanase was purified to electrophoretic homogeneity through Ni-NTA chromatography. After Ni-NTA purification, a 4.5-fold purification was achieved with 56% recovery. The purified rPoMan5A showed a single band with an apparent molecular mass of 61.6 kDa by SDS-PAGE (Figure 3).

### Biochemical properties of the purified recombinant endo-1,4-β-mannanase

#### Effect of temperature and pH on rPoMan5A activity and stability

**Figure 4:**
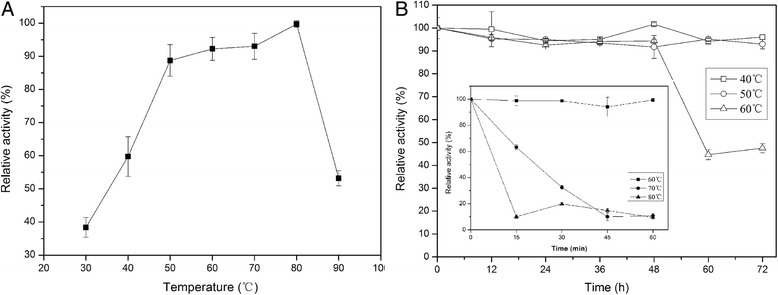
**Optimal temperature (A) and thermostability (B) of purified rPoMan5A.** The optimal temperature for endo-β-1, 4-mannosidase activity was measured at different temperatures using 0.5% locust bean gum as the substrate. The thermal stability was evaluated as the relative residual activity after incubation without the substrate at different temperatures at pH 4.0. Data are the mean of three replicates, and bars indicate the standard deviation of three replicates.

**Figure 5:**
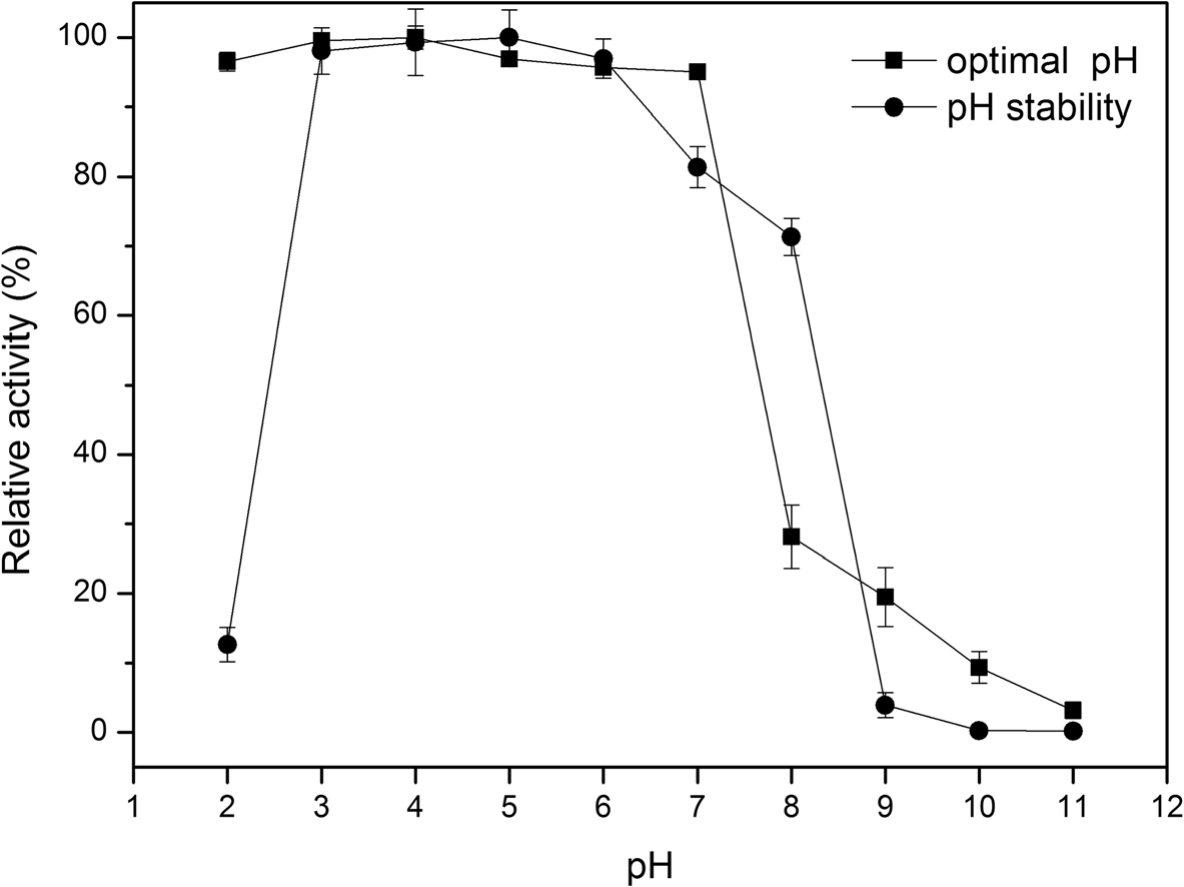
**Optimal pH and pH stability of purified rPoMan5A.** The influence of pH on enzyme activity was determined in different 50 mM buffers using 0.5% locust bean gum as the substrate. The pH stability was shown as the remaining activity after incubation for 30 min at 60°C in buffers at various pH without the substrate. Data are the mean of three replicates, and bars indicate standard deviation of three replicates.

The effects of temperature and pH on rPoMan5A activity were investigated using locust bean gum as the substrate. The rPoMan5A showed optimal activity at the temperature of 80°C and pH 3.0-4.0 and exhibited over 50% activity in temperatures ranging from 40°C to 90°C (Figure 4A). The thermal stability profiles showed that rPoMan5A was stable at temperatures below 70°C (Figure 4B). Almost no enzyme activity (>98%) was lost after 1 h incubation at 60°C, and the half-life at this temperature was 58 h. At 70°C, the half-life of the enzyme was only 21 min. The rPoMan5A retained more than 96% of its maximal activity at pH 2.0 (Figure 5). The pH stability profiles showed that the enzyme was highly stable within a broad range of pH values, ranging from 3.0 to 7.0 (Figure 5).

#### Effect of metal ions and chemical reagents on endo-1,4-β-mannanase activity

The presence of most metal ions at 1 mM or 10 mM (Ca^2+^, Cd^2+^, Cr^3+^, Co^2+^, Ba^2+^, Mg^2+^, Mn^2+^, Ni^2+^, Li^+^ and Fe^2+^) had little or no effect on the activity of rPoMan5A. At the high concentrations of Cu^2+^ and Fe^3+^ (10 mM), the activity of rPoMan5A was reduced to almost 50%. The whole activity of rPoMan5A was lost in the presence of 10 mM Hg^2+^ (Table 1). β-Mercaptoethanol (1 mM, 76.2%), dithiothreitol (DTT) (1 mM, 81.2%) and 0.1% SDS (92.0%) partially influenced the rPoMan5A activity, while 0.1% Triton-X 100 strongly decreased the rPoMan5A activity (25%).

**Table 1:** **Effects of metal ions on the activity of the rPoMan5A**

**Metal ions**	**Relative activity (%)**	
	**1 mM**	**10 mM**
Control	100.0 ± 2.7	
Ca^2+^	95.2 ± 0.3	98.6 ± 0.9
Cd^2+^	97.2 ± 0.7	105.1 ± 2.7
Cr^3+^	100.9 ± 0.3	101.1 ± 0.3
Co^2+^	100.0 ± 1.4	94.0 ± 0.2
Cu^2+^	86.5 ± 3.0	57.1 ± 3.8
Ba^2+^	98.6 ± 1.7	104.6 ± 0.9
Mg^2+^	97.6 ± 0.2	103.6 ± 0.8
Mn^2+^	85.4 ± 1.3	99.6 ± 2.4
Ni^2+^	95.4 ± 1.5	98.7 ± 5.6
Li^+^	97.1 ± 1.0	105.7 ± 3.2
Fe^2+^	97.1 ± 1.0	103.5 ± 2.0
Fe^3+^	64.9 ± 3.3	50.0 ± 2.8
Hg^2+^	64.9 ± 3.3	0.0 ± 0.0

Data are the mean ± standard deviation of three repeats.

#### Substrate specificity and kinetics parameters

The rPoMan5A exhibited the highest activity with locust bean gum (100%) and lower activity for guar gum (30.0%). There was no enzyme activity with sodium carboxymethyl cellulose, filter paper, and beechwood xylan as substrate. The *K*_*m*_ and *V*_*max*_ values of purified rPoMan5A were 7.6 mg mL^−1^ and 1425.4 U mg^−1^ for locust bean gum, 2.1 mg mL^−1^ and 154.8 U mg^−1^ for konjac glucomannan, and 2.3 mg mL^−1^ 18.9 U mg^−1^ for guar gum, respectively (Table 2).

**Table 2:** **Kinetic parameters of rPoMan5A for the hydrolysis of various mannans**

**Substrate**	***V***_***max***_ **(U mg** ^**−1**^ **)**	***K***_***m***_ **(mg mL** ^**−1**^ **)**	**k**_**cat**_ **(s** ^**−1**^ **)**	**k**_**cat**_ **/** ***K***_***m***_ **(mL mg** ^**−1**^ **s** ^**−1**^ **)**
Locust bean gum	1425.5	7.6	1463.5	192.6
Konjac glucomannan	154.8	2.1	158.9	75.7
Guar gum	18.9	2.3	19.4	8.4

#### Analysis of the hydrolysis products

**Figure 6:**
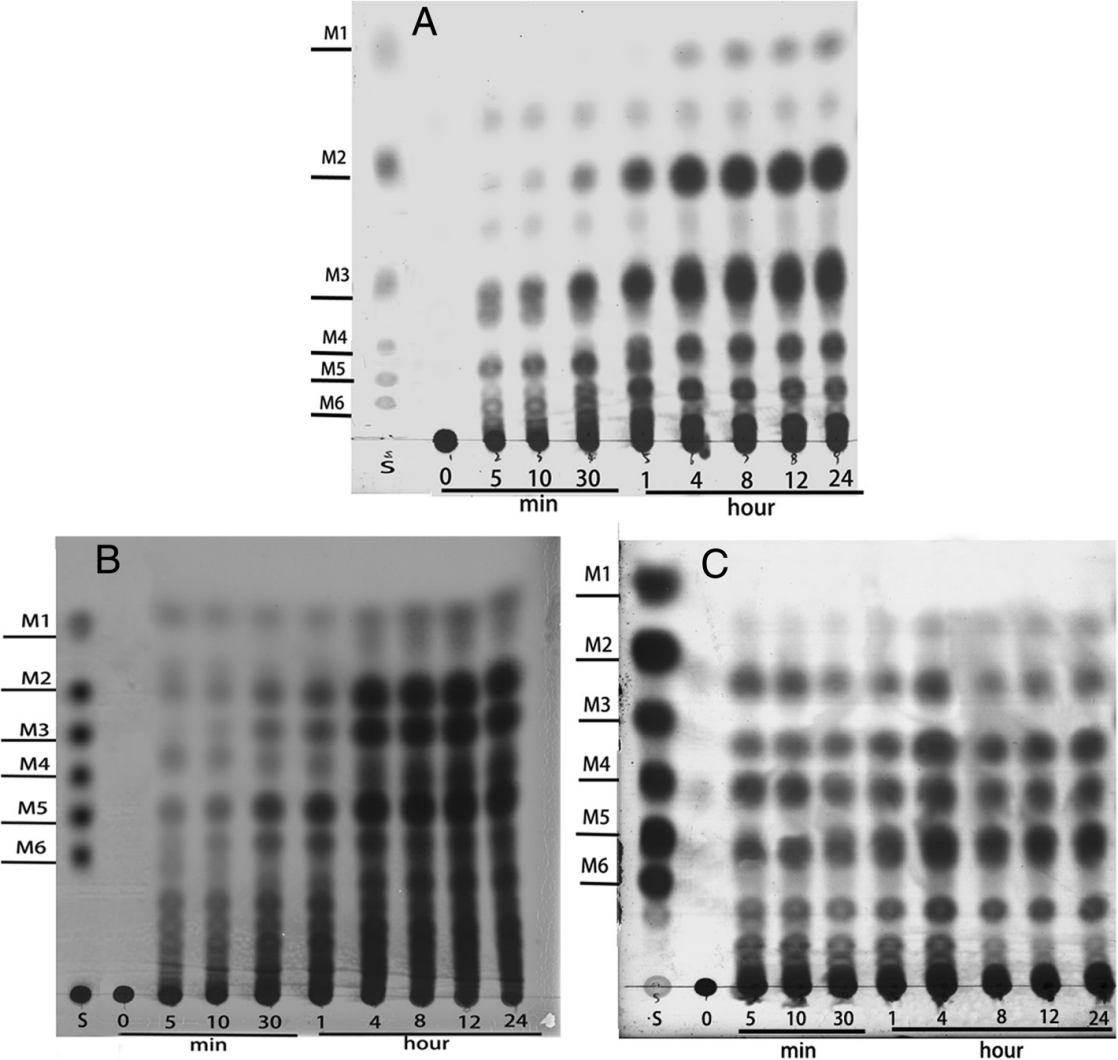
**TLC analysis of the products after hydrolysis of various mannan polymers (0.5%) by rPoMan5A.** The purified enzyme rPoMan5A and 0.5% various substrates (**A**: locust bean gum, **B**: konjac glucomannan, **C**: guar gum) were incubated in 50 mM acetate buffer (pH 4.0) at 60°C. Incubation times (hour or minute) are indicated. S, mannose and manno-oligosaccharides were used as standards.

The hydrolysis products of locust bean gum, konjac glucomannan, and guar gum by rPoMan5A were analyzed by thin-layer chromatography (TLC). As shown in Figure 6, the rPoMan5A hydrolyzed various types of mannan polymers, such as galacto-and glucomannans and released various mannose and manno-oligosaccharides. The hydrolysis of locust bean gum yielded mannobiose (M2), mannotriose (M3), and mannopentaose (M5), and other unidentifiable oligosaccharides as the main products (Figure 6A). The degradation of konjac glucomannan resulted in the formation of a mixture of mannose and manno-oligosaccharides (Figure 6B). Mannotriose (M3), mannotetraose (M4), and mannopentaose (M5) were the major products of hydrolysis of guar gum by rPoMan5A (Figure 6C). The release of mannose required at least 4 hours when locust bean gum were used as the substrate, but for konjac glucomannan and guar gum, there is already slight band after 5 min.

## Discussion

Our previous study demonstrated that the *P. oxalicum* GZ-2 is a robust hemicellulase-producing fungus during submerged fermentation used agricultural residues as carbon source [13]. In this study, a new endo-1,4-β-mannanase gene from *P. oxalicum* GZ-2 was cloned and successfully expressed in *Pichia pastoris*. The gene sequence of endo-1,4-β-mannosidase showed highest sequence similar (74%) to mannanase from *Aspergillus aculeatus* base on BLAST on NCBI (http://blast.ncbi.nlm.nih.gov/Blast.cgi). The amino acid sequence of endo-1,4-β-mannanase from *P. oxalicum* GZ-2 has 100% identity with the putative endo-1,4-β-mannanase from *Penicillium oxalicum* 114-2, followed by 71% with the likely endo-1,4-β-mannosidase from the *Penicillium roqueforti*. An abundance of endo-1,4-β-mannanases from other fungi genera such as *Aspergillus* and *Trichoderma* have previously been cloned and expressed in *Pichia pastoris* [5,7,14,15]. However, only few endo-1,4-β-mannanases from *Penicillium* genera have been cloned and expressed to date [16–18].

Based on amino acid sequence similarities, endo-1,4-β-mannanase has been classified into three families of glycoside hydrolases (family 5, 26, and 113, http://www.cazy.org). Most of fungal β-mannanases are predominantly belonging to family 5, and the bacterial β-mannanases belong to GH 5 or GH 26 [17]. Homology amino acid sequence comparison with other known fungal GH 5 β-mannanases showed that several catalytic active-sites are highly conserved. These features demonstrate that the rPoMan5A is a member of the GH5. A cellulose-binding domain (CBD) was identified in rPoMan5A at the N-terminus when aligned with available protein sequences from the NCBI databases. In GH5 fungal endo-1,4-β-mannanases, a CBD has been found at either the C-, or N-terminus of some enzymes that contained a catalytic domain (CD). For example, the CBD of *Trichoderma reesei* endo-1,4-β-mannanase (amino acid position 373-410) [19] and *Phanerochaete chrysosporium* (amino acid position 25-53) [20] are located at the C terminus and N terminus, respectively. The CBD play a very important role in the endo-1,4-β-mannanases activity. The CBD of *Trichoderma reesei* endo-1,4-β-mannanase has the ability to bind cellulose and increase the hydrolysis rate [21]. The endo-1,4-β-mannanase of *Aspergillus usamii* YL-01-78 consists of only a catalytic domain (CD), while a fusion of CBD from the *Trichoderma reesei* cellobiohydrolase into this enzyme could improve its catalytic activity and/or thermostability [22]. However, the mechanism of CBDs in catalysis still needs to be explored.

When the recombinant yeast was incubated at 28°C in a 1 L shake flask culture, a high level expression of endo-1,4-β-mannanase was obtained in *Pichia pastoris* (262 mg L^−1^). In comparison with other fungal endo-1,4-β-mannanase expression levels in yeast, like *Bispora* sp. MEY-1 MAN5A expressed 148 mg L^−1^ [23], *A. niger* endo-1,4-β-mannanase expressed 243 mg mL^−1^ [5], and *Trichoderma reesei* endo-1,4-β-mannanase expressed 150 mg L^−1^ [19], the yield of rPoMan5A was superior. The crude supernatant of *Pichia pastoris* showed good activity (84.4 U mL^−1^) and high specific activity (420.9 U mg^−1^), as well as high purity of the crude enzymes. Since cultivation experiments were performed in shake flasks which more or less limited the growth of yeast, it will result in considerably higher enzyme yields in a fermenter under controlled and optimized conditions [24]. Therefore, those features suggested the enzyme has great potential for large-scale production.

The purified rPoMan5A showed a single 61.6 kDa protein band on an SDS-PAGE (Figure 3), higher than that of the calculated molecular mass of the mature peptide (45.8 kDa). This may be due to the glycosylation of the expressed protein in *Pichia pastoris*. Two putative glycosylation sites in the PoMan5A amino acid sequence (^127^NFTI and ^229^NWSD) were predicted by the NetNGlyc 1.0 Server (http://www.cbs.dtu.dk/services/NetNGlyc/). The decrease in the molecular mass of rPoMan5A was verified by deglycosylation with Endo H_f_ on an SDS-PAGE (Figure 3). Many researchers had observed that the glycosylation of recombinant endo-1,4-β-mannanase in *Pichia pastoris* was able to obviously increase the molecular mass, such as endo-1,4-β-mannanase from *Aspergillus niger* BK01 [5], Man5C1 from *Penicillium pinophilum* C1 [16], and Anman5A from *Aspergillus niger* LW-1 [7].

The optimal temperature and pH for the rPoMan5A activity was 80°C and pH 3.0-4.0, respectively. For most fungal endo-1,4-β-mannanases the optimal temperature and pH are between 40-70°C and pH 4.0-6.0, respectively [25], such as endo-1,4-β-mannanase from *Rhizomucor miehei* (optimal temperature 55°C and pH 7.0) [26], MANI and MANII from *Aspergillus fumigatus* IMI 385708 (60°C and pH 4.5) [27], endo-1,4-β-mannanase from *P. oxalicum* SO (60°C and pH 5.0) [28], endo-1,4-β-mannanase from *A. niger* BK01 (80°C and pH 4.5) [5], and rMan5C1 from *P. pinophilum* C1 (70°C and pH 4.0) [16]. However, the rPoMan5A possesses some superior properties compared to these fungal endo-1,4-β-mannanases, such as a long half-life (58 h) at high temperatures (60°C) and a wide range of pH adaptability (pH 2.0-7.0) and stability (pH 3.0-7.0). Most fungal endo-1,4-β-mannanases were stable at temperatures below 50°C, such as rMan5F63 from *Penicillium freii* F63 (half-life of 30 min at 60°C) [18], recombinant man5XZ7 from thermophilic fungus *Thielavia arenaria* XZ7 (half-life of 30 min at 60°C) [29], and recombinant Man5XZ3 from *Aspergillus nidulans* XZ3 (half-life of 240 min at 60°C). Although rPoMan5A was unstable (less than 13% residual activity after incubation 30 min at pH 2.0) at pH 2.0, it exhibited 96.3% activity at such low pH (10 min assay). In comparison, the endo-1,4-β-mannanase from *Aspergillus niger* BK01 showed only 20% activity at pH 2.5 [5], and rMan5C1 from *P. pinophilum* C1 likewise lost 80% activity at pH 2.0 [16]. Many industrial bioprocesses involving enzymes depend on the use of low pH conditions to prevent contamination by other microbes. Therefore, rPoMan5A has the potential to be used in such processes, where fermentation requires low pH conditions. At its optimal catalytic temperature (80°C), rPoMan5A was unstable. This characteristic is similar to many other reported fungal endo-1,4-β-mannanases [18]. The enzyme binding to the substrate could partially stabilize the enzyme conformation, and having no substrate to bind resulted in the loss of enzymatic activity during a thermostability assay [16]. Therefore, endo-1,4-β-mannanase showed acidophilic and thermostable characteristics, features that not only increase the rate of hydrolysis but also reduce cost of production in the food industry. Consistent with our results, Hg^2+^ has been shown to strongly inhibit other endo-1,4-β-mannanases [7,26]. Inhibition of rPoMan5A activity by Hg^2+^ may be due to the oxidation of specific residues containing sulfhydryl groups such as cysteine. The mature protein of rPoMan5A has 10 cysteine residues. The influence of Fe^3+^ on rPoMan5A is similar to other endo-1,4-β-mannanases [15,30]. The activity of rPoMan5A in 1 mM reducing agents such as β-Mercaptoethanol and DTT as well as 0.1% SDS remained above 75%. SDS has been shown to almost completely inhibit endo-1,4-β-mannanase activity [11,16,23].

The rPoMan5A exhibited different activity against various substrates, such as locust bean gum (100% activity), konjac glucomannan (73.6% activity), and guar gum (30.0% activity). RmMan5A from *Rhizomucor miehei* displayed a same activity order: locust bean gum > konjac glucomannan > guar gum, when used to hydrolyze various mannans [26]. Locust bean gum is a galactomannan with a high mannose/galactose ratio of 4:1; konjac glucomannan is a glucomannan containing a mannose/glucose ratio of 3:1 and guar gum is a galactomannan with mannose/galactose ratio of 2:1 [31]. The structural differences between these three mannan polymers may lead to various enzymatic activities, when they are used as substrates. There was no enzyme activity detected on (hemi) cellulosic substrates, such as beechwood xylan, CMC-Na, and filter paper. The cellulase-free characteristic of rPoMan5A is in agreement with the most of fungal endo-1,4-β-mannanases [5,26,29]. The cellulase-free characteristic of rPoMan5A is valuable for the applications involving bleaching pulp and paper [10]. The *K*_*m*_ value of rPoMan5A for locust bean gum (7.6 mg mL^−1^) was close to the values obtained for some other fungal endo-1,4-β-mannanases, such as rMan5F63 from *Penicillium freii* F63 (7.8 mg mL^−1^) [18], rMan5C1 from *P. pinophilum* C1 (5.6 mg mL^−1^) [16], and Man5XZ7 from *Thielavia arenaria* XZ7 (5.3 mg mL^−1^) [29]. The *K*_*m*_ value of rPoMan5A for konjac glucomannan and guar gum was 3-fold lower than for locust bean gum.

Most endo-1,4-β-mannanases degrade the backbone of various mannan polysaccharides to produce primarily mannotriose and mannobiose with no mannose. For example, the hydrolysis by endo-1,4-β-mannanase from *Aspergillus niger* BK01 showed that mannobiose was the main product and mannose was not liberated using locust bean gum as substrate [5]. However, some endo-1,4-β-mannanases also released mannose from mannan polymers [18,23]. In this study, the endo-1,4-β-mannanases whether liberate mannose or not, were dependent on different substrates and hydrolysis time. Similar enzymatic properties were reported for RmMan5A from *Rhizomucor miehei* [26]. The action of the rPoMan5A on different substrates indicated that its function was not only as endo-β-1,4-mannanase but also as a 1,4-β-mannosidase. An abundance of manno-oligosaccharides primarily containing various oligosaccharides (M2 to M6) was obtained from hydrolyzing the inexpensive and widely available locust bean gum and konjac glucomannan by rPoMan5A. Because monogastric animals cannot effectively digest the mannans and galactomannans into nutritional components, the incorporation of endo-1,4-β-mannanase into their diets would help them to more easily digest the nutritional elements. Thus, rPoMan5A has a potential application in the food/feed industries. Although the enzyme rPoMan5A exhibited attractive biochemical properties like acid-resistant and thermostability, but there is need to explain the mechanisms of its superior properties. The molecular enzyme engineering should be considered to improve the catalytic efficiency and characteristics in the further studies.

## Conclusion

In this study, a new gene encoding a GH5 acidophilic thermostable endo-1,4-β-mannanase from *Penicillium oxalicum* was cloned and expressed in *Pichia pastoris*. High stability across a broad acidic pH range and thermostability make rPoMan5A better suited for many industrial applications in the food/feed and paper/pulp industries compared to most other fungal endo-1,4-β-mannanases.

## Methods

### Plasmids, strains and culture conditions

The *P. oxalicum* GZ-2 referred to in this study was isolated and identified as previously reported [13] and has been deposited at the China General Microbiological Culture Collection Center (CGMCC 7527). *Escherichia coli* strain TOP 10 (Invitrogen, San Diego, CA) grown in Luria Bertani (LB) medium at 37°C was used as the host cell for plasmid cloning and propagation of the recombinant expression pPICZαA vector. *Pichia pastoris* GS115 (Invitrogen, San Diego, CA) was used for heterologous protein expression. All media and protocols for *Pichia pastoris* are described in the *Pichia* expression manual (Invitrogen, San Diego, CA). Manno-oligosaccharides were purchased from Megazyme (Bray, Ireland). *P. oxalicum* GZ-2 was maintained and cultured according previously study [32]. *Pichia pastoris* GS115 was cultivated at 30°C in yeast extract peptone dextrose (YPD) medium (1% yeast extract, 2% tryptone, and 2% dextrose).

### Total RNA isolation, cDNA synthesis and cloning of the endo-1,4-β-mannanase gene

The *P. oxalicum* GZ-2 strain was grown at 30°C in a 250 mL shaker flask containing 50 mL basal culture medium in a shaking incubator using 2% corncob powder as the sole carbon source. After a 72 h growth period, the mycelium was harvested for total RNA isolation. The genomic DNA of *P. oxalicum* GZ-2 was extracted as described by Möller et al. [33]. RNA isolation and cDNA synthesis was performed as previously reported [32]. The degenerate primers manA-df and manA-dr were used (Table 3), which were designed using the iCODEHOP (http://blocks.fhcrc.org/codehop.html) program based on the multiple sequence alignment of homologous amino acid sequences using the ClustalW2 program. The partial sequence of *P. oxalicum* GZ-2 endo-1,4-β-mannanase gene was amplified by polymerase chain reaction (PCR) using the degenerate primers. To obtain the full-length gene, self-formed adaptor PCR (SEFA-PCR) was performed to amplify the 5’-end and the 3’-end of *poman5A* according to the protocol developed by Wang et al. [34] with the primers listed in Table 3. By aligning the sequences of the 5’-end and 3’-end PCR products, the full-length cDNA sequence of *poman5A* was deduced and obtained through RT-PCR using the following specific primers: manA-f and manA-r. The PCR fragment was purified and ligated into the pMD-19T vector and was designated pMD-man5A. The cDNA encoding the mature *P. oxalicum* GZ-2 endo-1,4-β-mannanase gene was PCR amplified with PrimeSTAR™ HS DNA Polymerase (Takara, Dalian, China) using the recombinant plasmid pMD-man5A as a template, with specific primers manA-ef including an *Eco*RI site, and manA-er including an *Xba*I site. After digestion with *Eco*RI and *Xba*I, The DNA insert was ligated into the pPICZαA vector downstream of the α-factor signal peptide sequence. Proper construction was confirmed by restriction digestion and DNA sequencing and was designated as pPIC-man5A. The nucleotide sequence of *poMan5A* has been deposited in the GeneBank under accession number KF233753.

**Table 3:** **Primers used in this study**

**Primer name**	**Primer purpose**	**Primer sequence**	**Size (bases)**
manA-df	Degenerate primers	CGGGTCTGGGGCTTYAAYGAYGT	23
manA-dr		GTG CCGTAGTAGATGGTRTTNCCRTCRT	28
manA-3-sp3	Amplify gene of the 3′-end by SEFA-PCR	ACTTTCCATCTGTACNNNNNNNNNTGTGAG	30
manA-3-sp2		TGACGACCGAGTCTGACGGAAGCTA	25
manA-3-sp1		CTGGGTCAAGGCGACTAGCCAATAT	25
manA-5-sp3	Amplify gene of the 5′-end by SEFA-PCR	GTGTTGATGGTGGCGNNNNNNNNNGCAAGA	30
manA-5-sp2		GGAGATGAGATGTACCGCGAAACCA	25
manA-5-sp1		CCATGTGCTTCGGGTCGAGTGATTT	25
manA-f	cDNA cloning primers	ATGACATTGGGATTGACTCAGACGA	25
manA-r		TCAGATCGCAGCGACATGATT	21
manA-ef	Specific expression primers	CGGAATTCCAGGTGGCGGAATATGGCCAGTGT	32
manA-er		GCTCTAGATAGATCGCAGCGACATGATTCGTCACCA	36
5′AOX	Confirmed primers	GACTGGTTCCAATTGACAAGC	21
3′AOX		GCAAATGGCATTCTGACATCC	21

R = A/G, W = A/T, Y = C/T, N = A/T/C/G.

### Expression of the endo-1,4-β-mannanase gene in *Pichia pastoris* in shaken flasks

The recombinant plasmid *pPIC-manA* was linearized with *Pme*I (New England BioLabs, Beverly, MA, USA), and then transformed into *Pichia pastoris* GS115 by electroporation (Gene Pulser Xcell™ Electroporation System, Bio-Rad, Hercules, CA, USA) according to the manufacturer’s instructions. The control was transformed with empty pPICZαA at same condition. The transformants were plated on YPDS plates (1% yeast extract, 2% tryptone, 2% dextrose, 1 M sorbitol, 100 μg mL^−1^ Zeocin, and 2% agar) and incubated at 30°C for 2-3 days until colonies appeared.

The colonies were first screened by selection on YPDZ (1% yeast extract, 2% tryptone, 2% dextrose, 2% agar) plates (Zeo1000) containing Zeocin at a final concentration of 1000 μg mL^−1^, then on YPDZ plates containing Zeocin at final concentrations of 2000 μg mL^−1^ (Zeo2000) to screen for multiple copies of a cDNA gene targeted gene [35]. The Mut^+^ phenotype of *Pichia pastoris* recombinants was confirmed by PCR with 5′AOX and 3′AOX primers using DNA from the *Pichia pastoris* transformant as the template that was extracted using a Yeast Genomic DNA Extraction kit (Tiangen, Beijing, China). Twenty colonies from the Zeo2000 plates were inoculated into 10 mL of YPM medium (1% yeast extract and 2% tryptone) containing 1% methanol in a 50 mL flask and cultured at 28°C on a rotary incubator at 250 rpm to induce the expression of the endo-1,4-β-mannanase gene. The transformant with the highest endo-1,4-β-mannanase activity in a culture supernatant following a 7 day incubation was used for further fermentation in 1 L flasks following the method of Bai et al. [36].

### Purification of recombinant endo-1,4-β-mannanase

All purification steps were performed at 4°C unless stated otherwise. The crude culture supernatant was harvested by centrifugation of the culture broth at 8000 rpm for 10 min at 4°C. The supernatant was subjected to precipitation at 80% ammonium sulfate saturation. After dialysis against 50 mM acetate buffer (pH 4.0), the proteins were collected and used for the next purification. The expressed 6 × His-tagged proteins were purified with Ni-NTA Sepharose (Qiagen, Valencia, CA) according to the manufacturer’s instructions.

### Enzyme activity and protein concentration assay

The enzyme activity was determined by the DNS method according to Miller [37] with some modifications. Briefly, 100 μL of enzyme diluted with 50 mM acetate buffer (pH 4.0) was added to 900 μL of 0.5% locust bean gum in 50 mM acetate buffer (pH 4.0) and incubated at 80°C for 10 min. The released reducing sugars were measured at 540 nm using D-mannose as the standard. One unit of endo-1,4-β-mannanase activity was defined as the amount of enzyme liberating 1 μmol of reducing sugars per minute under the conditions described above. Protein content was determined using a BCA protein assay kit (Dingguo Changsheng Biotechnology Co., Ltd, Beijing, China), and the standard curve was generated with bovine serum albumin (BSA) as the standard.

### SDS-PAGE and zymography analysis

Sodium dodecyl sulfate polyacrylamide gel electrophoresis (SDS-PAGE) was performed using a 12% (w/v) polyacrylamide gel with a 5% stacking gel and the Mini-Protean II system (BioRad) according to the method described by Laemmli [38]. The proteins were stained with Coomassie Brilliant Blue R-250 (Sigma Chemical, St. Louis, USA). The zymogram analysis was performed as previously reported by co-polymerizing 0.05% (w/v) locust bean gum with 12% polyacrylamide gel [13]. After the separation of the enzyme samples by SDS-PAGE, the gel was soaked for 1 h in 2.5% (v/v) Triton X-100 to remove the SDS and refold the proteins in the gel. The gel was then thoroughly washed three times in MilliQ water and incubated at 50°C for 60 min in 50 mM acetate buffer (pH 4.0). The gel was stained with 0.1% (w/v) Congo red solution for 30 min and destained with l M NaCl until pale-red hydrolysis zones were observed against a red background.

### Effect of temperature, pH and various reagents on enzyme activity

The optimal temperature for rPoMan5A activity was determined using the standard method as described above at various temperatures ranging from 30°C to 90°C. To estimate thermal stability, the diluted enzyme was pre-incubated in 50 mM acetate buffer (pH 4.0) at different temperatures (40-90°C) for various times in the absence of substrate and then assayed for remaining enzyme at 60°C using the standard DNS method.

For the estimation of optimal pH for rPoMan5A enzyme activity, 50 mM different buffer solutions like glycine-HCl buffer (pH 2.0-3.0), acetate buffer (pH 4.0-6.0), sodium phosphate buffer (pH 7.0-8.0), and glycine-NaOH buffer (pH 9.0-11.0) were used. The pH stability was determined at different pH-values for 30 min by incubation of diluted enzyme samples in the absence of substrate at 60°C. The residual enzyme activity was measured at 60°C for 10 min in the acetate buffer (pH 4.0) using the DNS method. The effect of metal ions on rPoMan5A activity was examined by incubating the enzyme with various metal ions (BaCl_2_, CuSO_4_, FeCl_3_, MnSO_4_, CaCl_2_, CdSO_4_, FeSO_4_, CrCl_3_, CoCl_2_, LiCl, NiSO_4_, MgSO_4_ and HgCl_2_) or reagents in 50 mM acetate buffer (pH 4.0) at 60°C for 10 min.

### Substrate specificity and kinetic parameters

The specific activity of rPoMan5A was measured using different substrates (0.5% (w/v), pH 4.0) in 50 mM acetate buffer (locust bean gum, konjac mannan, guar gum, sodium carboxymethyl cellulose (CMC-Na), filter paper, and beechwood xylan). To determine the reaction rate of rPoMan5A, various mannans substrate concentrations were used to react 10 min at 80°C in acetate buffer (pH 4.0, 50 mM). The Michaelis-Menten constant (*K*_*m*_) and the maximum velocity (*V*_*max*_) were calculated using Lineweaver-Burk plots.

### Thin-layer chromatography (TLC) analysis of the hydrolysis products

The analysis of hydrolysis products was performed by thin-layer chromatography using locust bean gum, konjac mannan and guar gum as substrates. The purified rPoMan5A (5 μg) was incubated in a reaction volume of 1 mL with 0.5% of substrate at 60°C in 50 mM acetate buffer (pH 4.0) for 24 h. Aliquots were collected at different time points and boiled for 10 min. All products were freeze-dried and dissolved in a methanol (70%). Then, 5 μL of each aliquot was spotted onto a silica plate (Merck, Germany, 10 × 20 cm), and the plates were developed twice with a solvent system consisted of chloroform-acetic acid-water (3:6:1, v/v) for 3-4 hours. Later, the plates were dried and hydrolysis products were detected by spraying with a 9:1 (v/v) mixture of methanol and sulfuric acid containing 0.2% orcinol and heating at 85°C for 5-10 min. Manno-oligosaccharides (mannose (M1), mannobiose (M2), mannotriose (M3), mannotetraose (M4), mannopentaose (M5), and mannohexaose (M6), Megazyme, Bray, Ireland) were used as standards.
